# An Analytical Compendium of the Various Branches of Medical Science, for the Use and Examination of Students

**Published:** 1848-11

**Authors:** 


*An Analytical Compendium of the various Branches of Medical Science, for the use and examination of Students.* By John Neill, M. D., Demonstrator of Anatomy in the University of Pennsylvania, Lecturer on Anatomy in the Medical Institutes [Institute] of Philadelphia, &c.; and Francis Gurney Smith, M. D., Lecturer on Physiology in the Philadelphia Association for Medical Instruction, Fellow of the College of Physicians, &c. Small 8vo. pp. 964. Philadelphia, Lea & Blanchard, 1848.

We do not share in the opinion entertained by some, that compendiums of science are not desirable, or with the still smaller number, who esteem them useless. On the contrary, when well executed, they are of essential service to the student; and, so far as we have seen, most of them have contained an amount of information, which older individuals—even they who disparage them—may be presumed to be far from possessing. Taking the work before us we can certainly say, that no one who has not occupied himself with the different scientific treatises and essays that have appeared recently, and has withal a rare memory, could pretend to possess the knowledge contained in it; and hence we can recommend it to such—as well as to students especially—for its general accuracy and adequacy for their purposes; and to the well-informed practitioner to aid him in recalling what may easily have passed from his remembrance. We regard such books as infinitely better than those on the "grinding" plan of question and answer; in which much space is taken up with the questions; whilst the answers must necessarily be brief. Their whole arrangement is, indeed, adapted rather for *cramming* the superficial than for conveying expanded information. But even those we do not consider useless books; and, instead of being fastidious as critics on such productions, we are really thankful to their authors, who have bestowed so much time and trouble upon them; for not one, perhaps, has appeared which has not been of some value in imparting knowledge to a greater or less number; and they who think otherwise had better refrain from purchasing them, rather than endeavour to prevent others from doing so. What may be useless to them, owing to the real or fancied extent of their information, may be of moment to others who are less endowed perhaps, but certainly more modest. There is a marked difference between a book that may be esteemed unnecessary, and one that teaches erroneous doctrines, and is therefore pernicious: the former may be tolerated, if not encouraged; whilst the latter ought to be discountenanced by all.

The authors of the "Compendium" before us are well adapted for the task they assumed. Dr. Neill is the Demonstrator of Anatomy in the University of Pennsylvania; and Dr. Smith is Lecturer on Physiology in the Philadelphia Association for Medical Instruction. The former, we have always heard, is a zealous and able teacher of practical anatomy, and has already published on his favourite branch: the latter, we know to be an accomplished gentleman; an assiduous instructor; and an active cultivator of the departments of medical science to which his attention has been especially directed.

The volume before us—the title of which is not, we think, the most classical—is formed of various "Handbooks"—to adopt an old Saxon and modern German word, which we prefer to "Manuals." We say the title is not the most classical. Passing by the typographical error which we have noticed in the heading to this article, we think the expression "for the use and examination of students" equivocal. The authors doubtless mean for the use of students, especially of those about to be examined; but the sentence means rather for the *use* of students and for the *examination* of students. But passing over this—what may be regarded hypercriticism—we may state that the separate "Handbooks" are, 1, On Anatomy; 2, On Physiology; 3, On Surgery; 4, On Obstetrics; 5, On Materia Medica and Therapeutics; 6, On Chemistry; and, 7, On the Practice of Medicine—the various branches, in short, that constitute the curriculum in our best medical schools. All these are bound together in the volume before us; but each may be purchased separately. They are richly illustrated by wood-cuts; and are carefully, and, generally, correctly, written. In one or two instances, we have noticed inadvertencies, or what strike us as such. For example, we do not clearly see what the author of the Handbook of Anatomy means at page 67 by the term "fasciculus."

"Every muscle," he says, "is composed of a number of bundles" [i.e. fasciculi] "of fibres, each of which consists of filaments; each filament is divided into fasciculi," [i.e. bundles] "and each fasciculus consists of a number of *primitive particles* or *sarcous elements*, held together by a tough, delicate, and elastic membrane called *sarcolemma*." p. 67.

And he elsewhere states,

"An anastomosis is the interchange of fasciculi between two trunks." p. 155.

In the Handbook of Materia Medica it was, of course, convenient—indeed, necessary—to adopt some classification. We have never placed much stress on any one in particular; but agree with the author in preferring a physiological arrangement. We are by no means, however, satisfied with the details of the one he has adopted, which is essentially that of Dr. Pereira. We may instance the first class of *cerebro-spinants ;* of which he makes five orders—1, Paralysers; 2, Convulsives; 3, Stupefacients; 4, Sedative stupefacients; and 5, Delirifacients—which we have always regarded, no matter by whom made—as calculated to force remedial agents into unnatural and therefore unjustifiable positions. The same applies to the second class *Stimulants*, of which he has two orders—1, Antispasmodics or Nervous Stimulants—as if all so *called* antispasmodics—for we deny that there is such a *thing* as a *direct* antispasmodic—must be necessarily nervous stimulants; and 2, Arterial Stimulants, in which all so called "stimulants" or "excitants" are comprised.

But if no other objection were applicable to the classification on which we are animadverting, it would be that three of the most potent articles of the materia medica—mercury, iodine and arsenic—are at the very end as "medicines not classified," whilst other agents that are equally considered to act upon the system of nutrition are placed under other heads; sarsaparilla, for example, under that of Diaphoretics! The author of the Handbook, however, is only to blame—if blame is to be attached to him—for having followed others in what we think is "more honoured in the breach than the observance."

We repeat our favourable impression as to the value of this book, or series of books: and recommend it as decidedly useful—to those especially who are commencing the study of their profession. We are confident that it will lead them safely in the true path—certainly on almost all the topics embraced by it.

